# The economic circumstances of widows in Vietnam

**DOI:** 10.1371/journal.pone.0285595

**Published:** 2023-05-10

**Authors:** Duc Hong Vo, Chi Minh Ho, Anh The Vo

**Affiliations:** The CBER–Research Centre in Business, Economics & Resources Ho Chi Minh City Open University, Ho Chi Minh, Vietnam; The Australian National University, AUSTRALIA

## Abstract

Information on the living conditions of widows in Vietnam is limited. Prior studies of gender gaps have identified widows as vulnerable to the risks of poverty. However, widows are only included as a sub-group in broader studies of women’s well-being. Large gaps remain in the knowledge based on the factors affecting both widows’ susceptibility to poverty and the conditions or circumstances that might minimize these risks. This paper attempts to help redress these knowledge gaps by contributing an analysis of data from the 2018 Vietnam Household Living Standard Surveys. The paper compares the likelihood of poverty for widowed and other households using probit regression. It also assesses whether widows who head up their households face different risks of poverty from those who live in other households. Finally, the paper examines the effects on the risk of poverty of a range of social, demographic and locational characteristics of widow households in Vietnam. Our results indicate that widowed households have experienced a higher probability of falling into poverty. Moreover, widow-headed households have faced more vulnerability to fresh water and housing, especially among widowed households. Policy implications have emerged based on the findings of this paper.

## 1. Introduction

Vietnam is also somewhat unique in the range of state institutions that have been explicitly established to advance the position of women in terms of their political representation. Women’s involvement in decision-making and the paid workforce is higher in Vietnam than in comparable countries. Thus, it is commonly claimed that Vietnam has already achieved gender equality. However, some commentators question the gaps between traditions or laws on the one hand and practices on the other [1].

Vietnam is also a rapidly growing economy with an emerging cohort of ‘gender specialists’ and a growing literature on gender inequality [2]. [2] are concerned about the persistence of gender inequalities in access to resources such as education, services, credit and land. However, the authors also note that most studies have been descriptive, and there is still a lack of sophisticated quantitative and qualitative analyses. Thus, there is still a need for research, development activities, and curriculum materials addressing gender issues in Vietnam as it grows in economic and political importance.

A limited number of academic studies [3–5] has focused on intra-gender differences (i.e., within-a-single gender), in which the inequality among female-headed households (FHHs) has not been systematically investigated, particularly in the Vietnamese context. Among FHHs, widows are an important sub-group commonly viewed as amongst the poorest of the poor [6]. There are some reliable reasons to expect that widows might be especially susceptible to poverty. The death of a household’s breadwinner will likely lead to a significant loss of income, which a surviving spouse might find difficult to replace [7–9]. Furthermore, the households most exposed to the risk of widowhood are, on average, those already most likely to have unfavourable economic characteristics, given the strong correlation between socio-economic status and life expectancy [10,11].

However, conflicting empirical evidence exists on the link between female household headship and poverty [6,12]. In any case, it may also be possible that the risks of poverty for widows are different from other FHHs. Widows’ route into household headship differs from that of, for example, women who are heads due to separation, divorce, or remaining single. Additionally, not all widows become household heads, with many women joining the household of other family members (such as a married son) when their partner dies [13]. Based on household income or expenditure measures, it is possible that the poverty risks for widows in these latter circumstances might be lower than for other widows and FHHs.

The task of enquiring into which widows are exposed to poverty and why is thus far from straightforward. It is important to avoid a ‘blanket generalization’ that a link between widowhood and poverty exists and that this link applies to all widows in all contexts. The extant literature [6,9,12–15] also highlights the importance of examining the diversity in widows’ risk of poverty, taking account of possible differences across groups of widows defined by whether they are a household head or not, the composition of their household in terms of, for example, the number and age of dependent children, the age of the woman herself, rural versus urban location, and more. It is also important to investigate poverty risks using a broad informational base that allows for the possibility that data on household income might poorly capture a widow’s well-being (and that of her children). This will be the case if a woman is living in a relatively affluent household (with high per-capita income) but, due to a lack of bargaining power within the household, she has little ability to control how resources are used [6,13]. It can also be the case that a woman in a less affluent household will achieve a high level of well-being if, by being the household head, she can achieve more independence and control over her life’s outcomes [16].

The Vietnamese culture has been heavily influenced by Confucian ideology. Widows have been discouraged from remarriage to be faithful to their deceased partner. More importantly, they are responsible for caring for the husband’s family [17]. Nowadays, family size in Vietnam tends to reduce, and the quantity of multi-generational families also declined toward migration and modernization [18,19]. Using the VHLSS 2018 data, we find that 12.1 per cent of our research sample are widow-headed households (WHHs). Among WHHs, 365 households live alone (equivalent to 32 per cent), and 257 WHHs live with one other adult (equivalent to 23 per cent), in which nearly half of these adults are dependent members who are household members but do not have a job, do not contribute to household income, and do not do housework. On the other hand, 23 per cent of WHHs are living with one child who is under 16 years. These statistics reveal that many WHHs live alone or reside with many dependent members, including children and adults who cannot make a living. Besides, in 2018, more than two third of the WHHs were living in rural areas. Particularly, 18.4 per cent of WHHs are classified as poor households. The figures indicate that key sources of poverty are associated with WHHs in Vietnam.

Because WHHs in Vietnam have somewhat unique characteristics, studies within this context can help highlight the diversity in such households and thus help counter the tendency to generalize their characteristics and outcomes. WHH is a relatively small group. While widowhood is exogenous with the household head’s decisions, it depresses the widow’s wellbeing. To this extent, WHHs in Vietnam may suffer a significant risk of poverty. However, the probability of WHHs falling into poverty has not been investigated in the current literature, particularly for emerging countries such as Vietnam. Studying widows’ experience of poverty in the Vietnamese context provides novel insights into whether ostensibly pro-women policies and institutions translate into favourable economic outcomes for key groups. This motivation warrants this study to examine the poverty risks for widows in the Vietnamese context.

Following this introduction, the remainder of this paper is structured as follows. Section 2 discusses and synthesizes relevant empirical studies highlighting the research gaps in the existing literature. Section 3 discusses data and research methodology. Empirical results are presented and discussed in section 4, followed by concluding remarks and policy implications in section 5 of the paper.

## 2. Literature review

### 2.1. Sources of poverty

There are four typical sources causing poverty, including regional characteristics, community characteristics, household characteristics, and individual characteristics [20]. The regional sources of poverty are related to country or regional characteristics, which are involved with macroeconomic factors and geographical and natural conditions. Ethnic minorities and regions which are isolated and are inhospitable climatic conditions tend to be easily vulnerable to poverty. Besides, poor infrastructure and lack of public service availability are also correlated with a high probability of poverty. Inversely, urban regions with high population density, the Mekong Delta and coastal regions with developed infrastructure have advantages to overcome poverty.

Regarding household characteristics, household size, employment status of household members, dependency ratio, and the head’s gender are considered significant determinants of household poverty [20]. A larger household size implies a higher efficiency in household expenditure toward the economy of scale. On the other hand, household income relatively depends on the quantity of economically active members in the household. As such, adding these economically active members to the household tends to increase household net income as per capita income is higher while per capita expenditure reduces. As a result, a higher proportion of households can avoid poverty. In contrast, dependent members who cannot work do share household expenditures. However, they do not earn income for the households. Ethically, dependent members are family members and shall be treated fairly like others. As such, the household size and the dependency ratio contribute to household wealth differently [21].

Besides, a recent study from [5] confirms the gender gap in household wealth in Vietnam. These authors also confirm the heterogeneous wealth across different marital statuses. Particularly, widowhood is a special marital status which is not controlled by the couple, while other family statuses, including single, married, separated or divorced, are. WHHs are in an exceptional situation where the head’s partner, who played a significant role in the household, passed away. Losing a member reduces income sources and the efficiency of numerous families resulting in many difficulties in financial balance in WHHs. However [5], did not particularly focus on the difficulties of WHHs.

### 2.2. The overviews of the economic circumstances of widows

Early studies have shown that widows face a high risk of poverty because of the loss of family wealth after their husband’s death. The death of a spouse can be a devastating and life-altering experience, and widows often face numerous challenges after their loss. These challenges include financial difficulties [7,22–26]. Besides, widowhood leads to higher poverty rates for all women, regardless of pension eligibility or annuity choice for retirement. This is because when a husband dies, much family wealth is lost, including private pension income. Also, the loss of wealth is not always compensated by other resources such as life insurance, and poor widows often had little housing wealth when they were married [7]. In their study regarding the economic situations of middle-aged and older widows [26], found that widows in these age ranges face fewer economic resources after their spouse’s death than their married counterparts. Recent research by [24,25] also confirms the negative impact of widowhood on household income. They conclude that the magnitude of the effect depends on individual and household characteristics as well as social and contextual factors.

A female’s loss of a spouse can also mean a loss of social support and the need to assume responsibilities previously handled by their spouse [27]. Thus, the labour participation of women is significantly affected by widowhood. [28] studies the impact of widowhood on Indian women’s labour force participation using panel data that tracks women before and after the event of widowhood. The results confirm a strong age pattern, where women widowed before the age of 52 saw an increase in the number of days worked, while women widowed after the age of 52 saw a reduction. In addition, widowed women are more likely to be employed in the non-agricultural sector. They have higher incomes than married women if they become the household head after their spouse’s death.

The economic gap between widows and married individuals may be due to the lower levels of financial literacy and experience in financial planning among women in general [25,29]. Therefore, couples need to prepare psychologically and have a clear retirement plan to avoid the risk of poverty after widowhood. Many studies found that the most significant risk of falling into poverty occurs in the initial stage of widowhood [23,25], highlighting the importance of having sufficient resources before retirement to decrease the chance of facing poverty in retired years and after the loss of a spouse.

Vietnamese studies that specifically address the economic circumstances of widows are rare, and typically, when widows are mentioned, it is only as part of a broader analysis of the situation of women. For example [30], includes widow households in his study of Vietnamese FHHs, and [31] mentions them in her study of the impact of lone motherhood on children’s education outcomes. [32] also include widows in their broad study of older persons in Myanmar, Vietnam, and Thailand, finding evidence that widows are more likely to live solo, which makes them more vulnerable to stress, disabilities, and financial pressure. [31]’s study adds evidence to the disadvantaged economic position of widows by showing that widowed lone mothers have a relatively high prevalence of disability, a relatively low level of educational attainment, and a relatively high level of economic activity. However, they mostly work in low-skilled sectors. [31]’s study also shows that children of widowed lone mothers have the lowest level of school performance, as measured by school enrolment and completion rates, pointing to concerning impacts of widowhood on the outcomes of both the widows themselves and their children.

Widows were also an important group included in [33]’s qualitative investigation of gender and land entitlements in northern Vietnam. This study provided more pointers to the possible sources of poverty among widows. It highlighted the barriers faced by widows in exercising their inheritance rights over land and other assets. Scott also found that widows faced barriers in accessing the information on land allocation. Thus, they commonly experienced difficulties acquiring land for production and as collateral to secure bank loans [33–35].

Recently [5,21], approached widowed households as a moderating factor while investigating a gender wealth gap and a wealth return on education in the Vietnamese context, respectively. In these recent studies, widowhood significantly and negatively impacts household wealth accumulation compared to other households with different marital statuses. Particularly, at the upper asset threshold (from 440.25 million VND in 2018) [21], found that wealth accumulation favours single households over widowed households. However, the authors found relatively weak differences in wealth accumulation between the two cohorts. On the other hand [5], found that WHHs consistently stay in the vulnerable group across all net worth quantiles.

To our knowledge, the only prior Vietnamese study with a primary focus on widows is [36], in which the authors use the 2009 census data. This study identified that widows who were relatively old, had a disability, or attained a relatively low education level were more likely to live independently. In addition, ethnicity was also identified as an important correlate of living on one’s own in widowhood, with women in minority groups more likely to be in this situation than other Vietnamese widows.

Our study adds to [36] analysis by focusing squarely on the factors linked to widows’ vulnerability to poverty and expanding the analysis to widows living in FHHs and other household situations. First, we compare the likelihood of poverty for widows and other Vietnamese women. We also assess whether widows who head up their households face different risks of poverty from those who live in other households. Finally, we examine the effect on the risk of poverty of a range of social, demographic and locational characteristics of widow households in Vietnam.

Our literature review indicates that selected social and economic aspects, such as the wealth and welfare of widows, have been investigated in developed countries [25,27,28]. In addition, efforts have been made to propose several solutions to mitigate poverty after widowhood [23,25,29]. However, widowed households suffering from poverty have largely been neglected in the existing literature, especially in Confucian countries such as Vietnam. This research gap warrants the analysis below to be conducted.

Barriers due to traditional Confucian norms prevent women, especially widows, from asset ownership leading to vulnerability. We employ the probit regression for various samples, including the entire sample, a sub-sample of widowed households where at least one widow is living, and a sub-sample of female-headed households to examine the probability of suffering from poverty of the widowed households. Besides, other proxies such as land value and income are also used as the alternative dependent variable to investigate the effects of widowhood on a household’s wealth [5]. We also extend our analysis by estimating the marginal effects of widowhood on several forms of insufficiency, including suffering from food, foodstuff, electricity, water, housing, and clothing. Our analytical approach is discussed in detail in the next section.

## 3. Data and research methodology

### 3.1 Data

This study utilizes data from the 2018 Vietnam Household Living Standard Survey (VHLSS2018) to examine the economic outcomes of widows in Vietnam and to assess the factors that influence their risk of poverty. The VHLSS has been conducted by the General Statistics Office of Vietnam since 2002 with technical assistance from the United Nations Development Programme and the World Bank [37]. It plays a key role in Vietnam’s attempts to monitor the living standards of Vietnamese households, and it is widely used to inform policy decision-making. Data from the VHLSS has been used to evaluate developmental programs associated with the Comprehensive Poverty Reduction and Growth Strategy, the Millennium Development Goals, and Vietnam’s socioeconomic development goals [38].

### 3.2 Measuring poverty

There are two typical kinds of poverty in the literature: (i) absolute poverty and (ii) relative or multi-dimensional poverty. Absolute poverty is usually measured by income. In this criteria, the poverty line is a benchmark to identify "a poor household". Besides the poverty line, the bottom 10 or 20 per cent of income is usually perceived as the ’a poor group’. However, assigning a proportion of households whose income is lower than the average or median income within the region is sensitive to personal preference. On the other hand, income-based measurement is typically underestimated.

Regarding the second kind of poverty measurement [39], argue that poverty is essentially the lack of the means to live. The fundamental of poverty relies on a serious problem of what is needed to live "a decent life" and, more critically, what it is to be human. From this perspective, multi-dimensional poverty is more appropriate because it captures many social-economic aspects (including incomes, social needs, and many others) to identify "a poor household" [40–43]. Therefore, measuring poverty by a constant poverty line or a proportion of income is inadequate.

In 2015, the Vietnamese government formally released official documents to identify a poor household by multi-dimensional measurements which are officially announced throughout the Decision number 59/2015/QD-TTg dated 19/11/2015 by the Prime Minister about the multi-dimensional poverty standards. There are two major pillars to assign whether a household is poor: (i) the income-based measurement and (ii) norms on deprivation of access to basic social services. *First*, regarding the income criteria, the Vietnamese government assigns a poor household based on monthly income. *Second*, regarding the norms on deprivation of access to basic social services, there are five categories (including (i) health; (ii) education; (iii) housing; (iv) clean water and sanitation; and (v) information), which are measured by ten indices (including (i) access to medical services; (ii) health insurance; (iii) education level of adults; (iv) school attendance of children; (v) housing quality; (vi) average housing area per capita; (vii) residential water sources; (viii) hygienic latrines and toilets; (ix) telecom services; and (x) assets to serve information access). The government also assigns different benchmarks of poverty in rural and urban areas. In the rural area, a poor household is identified if its monthly income is lower than 700 thousand VND or if its monthly income is from 700 thousand VND to 1 million VND, and the household fails in at least three indices of the second criterion as discussed above. In urban areas, the two milestones of monthly income are 900 thousand VND and 900 thousand VND to 1.3 million VND, respectively. The conditions of norms on deprivation of access to essential social services in rural and urban regions are identical. The VHLSS 2018 has a question to identify a poor household: “Have the local authorities classified your household as ‘poor’ in the commune/ward in 2018?”. As such, poor households have been classified by the local authorities. The local authorities have to strictly follow the official guidance from the prime minister’s decision to classify a poor household.

Based on the above ground, the multi-dimensional poverty measurement is appropriate to identify poverty. Besides, the process of identifying a poor household is strictly managed by the government authorities in the Vietnamese context. As such, we consider it appropriate to use the current identification of poor households in the VHLSS for our analysis.

### 3.3 The analytical framework

In the first part of our analysis, we examine whether Vietnamese households with a widow are more vulnerable to poverty than households that do not include a widow. Our key outcome measure is based on the responses to a VHLSS question: “Have the local authorities classified your household as ‘poor’ in the commune/ward in 2018?” In this sense, the outcome variable is binary, at which the value of “0” is proxied for non-poor households and the value of “1” denotes the poor household.

In further stages of our analysis, we extend the set of outcome variables to include household income and land ownership. We assess the household income and land holdings differences between the widow and non-widow households. These additions allow us to account for the possibility that there may be widow households that are not subject to poverty *per se* but still experience a relatively high level of economic hardship due to low income/wealth. It is important to identify these risks if they exist, especially when the poverty threshold is so low. Household income is measured as the total labour income of all household members, plus other income from non-labour sources, over the previous 12 months. Labour income comprises salaries, wages, cash, benefits, allowances and in-kind payments from all jobs. Other sources of household income include agricultural activities, non-agricultural activities, gifts and presents. Regarding land ownership, the VHLSS has a range of measures, including the size and value of each land plot owned by the household. In this study, we use the total value of all land plots owned within the household.

In each stage of our analysis, we incorporate controls to account for the likely influence of a range of individual and household characteristics on widows’ chances of poverty. We use VHLSS data on household size, the number of absent members, the household dependency ratio, and information on the household head’s characteristics, such as their level of education, age, injury status, and labour force participation status. These factors have been discussed as determinants of a household’s consumption and welfare by [44].

Following [44], we measure the household size by its equivalent scale adjustment, considering the number of individuals in the household, their age and likely household economies of scale. We measure the equivalent scale adjustment recommended by Deaton and Zaidi (2002):

$$EA={\left(A+\alpha C\right)}^{\theta }$$

where EA denotes the equivalent-adjusted household size; A represents the number of adults, and C is the number of children under the age of 15 years; *α*, which ranges from 0 to 1, represents the weighted expenditure requirement of a child relative to an adult, and *θ*, ranging from 0 to 1, measures the household economies of scale.

[44] argue that raising a child can be less costly in developing countries. The economies of scale can be larger because they spend a larger proportion of income on food (while maintaining a fixed expenditure). Thus, these authors suggest that in developing countries, *α* shall be low, in the range of 0.3–0.5, and *θ* shall be high, from 0.9 to 1. In addition, since 2002, Vietnam’s average income has increased substantially, and raising a child has also been more costly. For this reason, we use a value of *θ* = 0.9, which is less than 1, and *α* takes a value of 0.5 as the highest weight suggested by [44].

We measure absent household members as members who have not stayed in the household for at least six months. The dependency ratio measures the proportion of all household members that do not participate in the labour market, do not do housework and do not receive pensions, unemployment allowances, one-off severance pays, or an allowance for loss of working capacity the past twelve months. Finally, the injury variable identifies the total times household members have had such a severe injury that they have needed care or had to stop working, studying, or participating in other normal activities in the previous 12 months.

We also include locational variables to help control for spatial heterogeneity [45]. Of particular relevance in the current study is the relatively high rate of poverty in the Midlands and the Northern Mountainous Area, as well as in the Northern and central coastal and Highlands regions. The high prevalence of poverty in the Northern Mountainous Areas is partly due to its geography, which prevents the region from diversifying its economic activities, accessing major markets and improving its infrastructure. In addition, these northern regions are also the home of several ethnic minorities, among the poorest of the poor in Vietnam [46–48].

The regional breakdown of our data is eight-fold, comprising the Red River Delta, the Northern and Southern regions, the Highlands, and the Mekong Delta. An urban/rural categorization of locations identifies cities in Vietnam according to their official ranking, which reflects their function and role, the structure and level of their social-economic development, and their size. The highest-ranked cities (type I) have central roles in the social-economic development of their region and the nation. These cities also have the largest populations (more than 1,000,000 inhabitants), the highest population density (more than 2,000 inhabitants per square kilometre) and the highest proportion of non-agricultural labour (more than 65%). The cities of lower ranking perform less substantial political and economic roles and have lower populations and population densities.

### 3.4 Research methodology

In the above section, poverty has been described as a dummy variable at which “0” stands for a poor household, and “1” represents a non-poor household. Then, to analyze the vulnerability of widow households to poverty, probit regression and logit regression are the two appropriate methods for dealing with the binary dependent variable. On the one hand, logit regression can perform well regardless of whether the dependent variable is multinomial or binary. On the other hand, the probit regression mainly focuses on the binary dependent variable. In our study, both models are appropriate to analyze the probability of suffering poverty in widow households. As such, the probit regression is used to conduct our empirical analysis as the basis case, whereas logit regression is used as the robustness analysis. The empirical results of this robustness analysis are presented in the Appendix.

Derived from a latent variable model [49], the regression model takes the form:

$$y^{*}=\alpha Widowhh+\varepsilon$$

Where y* is a latent variable, the household is classified as poor when y*>0. Widowhh is a categorical variable identifying whether the household includes a widow, and *ε* is the error term (assumed to have a standard normal distribution where its cumulative density function is *f*(*x*)).

The probability of a household being classified as poor is defined as:

$$P(y^{*}>0|Widowhh)=P(\varepsilon >-\alpha Widowhh|Widowhh)=f(\alpha Widowhh)$$

Because the heterogeneity in household and individual characteristics can potentially explain differences in poverty status, we extend the probit model to include measures of these characteristics as control variables.


$$P(y^{*}>0|Widowhh,X^{\prime })=f(Widowhh,X^{\prime })$$

As noted, the control variables in *X*′ consist of: measures of household size, the number of absent household members, the household dependency ratio, the head’s age (including a squared term to account for possible non-linearity), the value of the land owned within the household, and locational and educational variables. Educational attainment is measured by years of education, based on the highest grade of education the person completed. Respondents who left school at Grade 12 are assigned 12 years of education, whilst those with a College, Bachelor, Master, or PhD degree are assigned 14, 15, 17, or 18 years of education, respectively. Urban location is measured with dummies for large and medium cities, with the rural location being the reference category. Also included are eight regional variables, with the Red River Delta being the reference category.

Because we are also interested in whether WHHs are more vulnerable to poverty than other widow households, we run a similar probit regression model on the VHLSS data, including variables to identify households headed by a widow (WHH) and, secondly, other widow households. The other elements of the model remain unchanged. To address whether widow-headed households are more susceptible to poverty than other female-headed households (FHHs), we apply the regression model to the sub-set of FHHs.

Our analysis of the correlation between widowhood and household income and land ownership deploys Ordinary Least Square (OLS) regression models, with both household income (*hhincome*) and the value of land owned by the household (*land*) logged to enable us to focus on relative differences where zero values are replaced with a number infinitesimally close to zero (e.i. 0.000000001). The models use the same key explanatory variable (*Widowhh*) as the probit models. The set of control variables is also similar. However, in the model of household income, land value is one of the control variables, whilst, in the land value model, household income is retained as a control variable.

In the final step, we examine the factors that affect widows’ relative chances of being exposed to different forms of poverty. Different probit regression models target food, housing, energy and other forms of insufficiency. The control variables identify household and individual characteristics, including the head’s education, age, injury status, whether he/she is participating in the labour force, and household size, location, and dependency ratio.

Summary statistics on the variables included in the regression models are provided in Table 1. These clearly show a relatively high incidence of poverty amongst WHHs. In all samples, Columns 2 and 3, 10.6 per cent of the households are considered poor. Within the sub-sample of the WHHs, 18.4 per cent of them is considered poor household, as presented in the last column. On the other end, for the widow households where the head is not a widow, results presented in columns 6 and 7 indicate that 16.1 per cent of the households are considered poor. In contrast, for the sub-sample of non-widow households, only 9.4 per cent are considered poor, as presented in columns 4 and 5.

**Table 1:** Descriptive statistics of Vietnamese households.

Variable	All sample		Non-widow households		Widow households		Widow-headed households	
(1)	(2)	(3)	(4)	(5)	(6)	(7)	(8)	(9)
	*number*	%	*number*	%	*number*	%	*number*	%
Number of households	9,399	100	7,723	100	1,676	100	1,134	100
Poor household	998	10.62	729	9.44	269	16.05	209	18.43
Food insufficient household	187	1.99	146	1.89	41	2.45	30	2.65
Foodstuff insufficient household	503	5.35	388	5.02	115	6.86	81	7.14
Electricity insufficient household	258	2.74	217	2.81	41	2.45	26	2.29
Water insufficient household	168	1.79	138	1.79	30	1.79	23	2.03
Housing insufficient household	342	3.64	288	3.73	54	3.22	38	3.35
Clothing insufficient household	299	3.18	240	3.11	59	3.52	37	3.26
Household living in the rural area	6,750	71.82	5,550	71.86	1,200	71.60	799	70.46
Household living in a medium city	1,482	15.77	1,213	15.71	269	16.05	185	16.31
Household living in a large city	1,482	15.77	960	12.43	207	12.35	150	13.23
Household living in Red River Delta	1,845	19.63	1,495	19.36	350	20.88	252	22.22
Households living in the Western North region	660	7.02	567	7.34	93	5.55	48	4.23
Households living in the Eastern North region	1,149	12.22	941	12.18	208	12.41	110	9.70
Households living in the North of the Middle region	978	10.41	792	10.26	186	11.10	112	9.88
Households living in the South of the Middle region	1,089	11.59	856	11.08	233	13.90	173	15.26
Household living in the Highland region	651	6.93	555	7.19	96	5.73	66	5.82
Households living in the Eastern South region	1,122	11.94	943	12.21	179	10.68	140	12.35
Household living in the Mekong Delta region	1,905	20.27	1,574	20.38	331	19.75	233	20.55
*Mean value*								
Household size (equivalent scale)	2.95		2.97		2.85		2.389	
Number of absent members	3.467		3.33		4.12		2.74	
Dependency ratio (equivalent scale)	1.03		0.99		1.17		0.91	
Household income (VND’000)	165,129		170,955		138,284		119,647	
The total value of land holdings (VND’000)	644,292		647,948		627,494		563,265	
Head’s wage (VND’000)	19,622		21,382		11,513		6,526	
Other members’ wages (VND’000)	29,374		29,544		28,592		29,330	
Other income sources (VND’000)	116,132		120,028		98,178		83,790	
Head’s educational grade	7.49		7.85		5.87		4.75	
Head’s age	50.74		49.05		58.52		64.74	
Total injury times	0.39		0.37		0.44		0.37	

As could be expected, the heads of WHHs are relatively old. The average age of household heads in this group is 64.7 years, while in the broader group of widow households, the average age of heads is 58.5 years. In non-widow households, the average head age is 49.1 years. The head of widow-headed households also has a relatively low level of educational attainment. The average years of education for widows who are household heads are only 4.7, while heads in the broader group of widow households have, on average, 5.9 years of education. On average, the heads of non-widow households have had 7.8 years of education.

Widows who are household heads also have a relatively low average level of labour income. As a result, these households tend to be more reliant on the labour income of other household members. The average annual labour income of the heads of widowed-headed households is 6.5 million VND, and the average earnings of other household members in these households is 29.3 million. In comparison, in non-widow households, the average annual earnings of household heads are 21.4 VND million, and other members’ earnings only reach 29.5 VND million.

Pointing to the possible role of assets in determining the risk of poverty, non-labour income in WHHs is also relatively low (83.8 VND million per annum, compared to 120 million VND in non-widow households). The data in Table 1 show that WHHs appear to be most susceptible to food and foodstuff poverty, with this rate reaching 2.6% and 7.1% compared to the average rate in other households of 2% and 5.6%, respectively. The incidence of other forms of poverty (clothing, electricity, housing) in WHHs is generally similar to that experienced in the other groups.

## 4. Empirical results and discussions

We commence our presentation of the results with the estimates of the relationships between the risk of *household poverty* and various economic and demographic factors across widows and other households. Table 2 reports the marginal effects and robust standard errors of variables in four regression models of the probability of household poverty. The key variables of interest identify whether a widow is present in the household (Widowhh) and whether the household head is a widow (WHH). Column 1 presents the model results that examine whether the poverty risk for widow households is different from all other Vietnamese households. Column 2 has the results from the model that isolates the relative risk of poverty in two groups of widow households–those headed by a widow and those headed by someone else. Column 3 focuses on the possible differences in poverty risks between WHHs and other widow households. Column 4 presents results from a model designed to examine whether WHHs have different poverty risks to other FHHs.

**Table 2:** Determinants of household poverty, all Vietnamese households, widow households and female-headed households, probit marginal effects.

Variables	All sample	All sample	Widow Households^1^	Female-headed households^2^
Widowed household	0.025***			
	(0.007)			
Widow headed household		0.028***	0.034	0.019
		(0.009)	(0.022)	(0.016)
Widow household where the head is not a widow		0.020		
		(0.013)		
Household size (Equivalent scale)	-0.013***	-0.012***	-0.031***	-0.025***
	(0.003)	(0.003)	(0.009)	(0.006)
Number of absent members	0.004	0.004	0.041*	0.038**
	(0.009)	(0.009)	(0.022)	(0.017)
Dependency ratio (Equivalent scale)	0.052***	0.052***	0.001	0.033
	(0.014)	(0.015)	(0.029)	(0.024)
Head’s educational attainment	-0.016***	-0.016***	-0.021***	-0.020***
	(0.001)	(0.001)	(0.003)	(0.002)
Head’s age	-0.006***	-0.006***	-0.009***	-0.001
	(0.001)	(0.001)	(0.003)	(0.003)
Head’s age squared	0.000***	0.000***	0.000**	0.000
	(0.000)	(0.000)	(0.000)	(0.000)
Total injury times	0.007***	0.007***	0.008	0.009**
	(0.002)	(0.002)	(0.006)	(0.004)
Land value (Log)	-0.004***	-0.004***	-0.005***	-0.004***
	(0.000)	(0.000)	(0.001)	(0.001)
Large City^a^	-0.074***	-0.074***	-0.047	-0.072***
	(0.014)	(0.015)	(0.031)	(0.024)
Medium City	-0.048***	-0.049***	-0.029	-0.054***
	(0.009)	(0.009)	(0.022)	(0.018)
Western North^b^	0.084***	0.085***	0.069**	0.053**
	(0.012)	(0.012)	(0.034)	(0.026)
Eastern North	0.066***	0.067***	0.068**	0.039*
	(0.011)	(0.011)	(0.028)	(0.024)
North of the Middle	0.060***	0.061***	0.100***	0.077***
	(0.011)	(0.011)	(0.028)	(0.023)
South of the Middle	0.009	0.009	0.001	-0.024
	(0.012)	(0.012)	(0.027)	(0.023)
Highland	0.032**	0.032**	0.001	0.013
	(0.012)	(0.013)	(0.035)	(0.027)
Eastern South	-0.041***	-0.041***	-0.066*	-0.076***
	(0.014)	(0.014)	(0.034)	(0.026)
Mekong Delta	-0.018	-0.018	-0.024	-0.064***
	(0.011)	(0.011)	(0.027)	(0.022)
Observations	9,399	9,399	1,676	2,399

Note: a) living in the rural area is the reference category; b) Red River Delta is the reference category; Robust standard errors in parentheses; ***, **, * is 1%, 5%, and 10% significant level, respectively.

^1^ is a sub-sample of widow households where at least a widow lives.

^2^ is a sub-sample of female-headed households.

The results in Column 1 of Table 2 show that households where a widow is present, have, at mean values, a risk of poverty that is 2.5 percentage points higher than other Vietnamese households. The figures in Column 2 reveal that this risk differential applies especially to WHHs, who have a risk of poverty 2.8 percentage points higher, at mean values, than non-widow households. The risk of poverty in households that include a widow who is not the household head is also higher, but the gap is not statistically significant. The results in Column 3 show that, in the presence of controls for a range of economic and other characteristics, the risk of poverty in WHHs and other widow households is similar. The results in Column 4 reveal, further, that the poverty risks of WHHs are similar to those of other FHHs.

The results in the remainder of Table 2 help to identify some of the risk factors for poverty in widow households and other household types. Larger household size appears to be a protective factor, reducing the odds of poverty, especially in widow households. For example, across all Vietnamese households, an increase in household size by one reduces the chances that the household will be poor by 1.3 percentage points. However, in the sub-sample of widow households, a similar change in household size reduces the chance of poverty by 3.1 percentage points. This pattern of results implies that widows’ chances of poverty are relatively strongly affected by whether other adults are in their household. Moreover, it speaks to a relatively high economic dependence of widows on the contributions of other household members.

Notably, whilst a higher dependency ratio tends to be positively related to a household’s chances of poverty (Columns 1 and 2), this factor is less important in widow households (Column 3). Across the sample of Vietnamese households as a whole, one point rise in the dependency ratio is associated with a substantial 5.2 percentage point increase in the risk of poverty. However, in the sample of widow households (Column 3), this factor is not a statistically significant source of variation in the risk of poverty. This result is intriguing. In the broad sample, it could be the case that households are more susceptible to poverty if/when an adult member within the household is not economically active. The results differ from [5], in which the dependency ratio is associated with the wealthier household, especially at the top of wealth distribution.

In widow households, the dependency ratio–of differences in members’ economic participation—is less likely to be at play, and, thus, the dependency ratio is less consequential to poverty risks. From the VHLSS 2018 data, we find that in a part of WHHs–who live with at least one dependent member in the household, 82.8 per cent of these households have one dependent adult and up to three children. These figures indicate that a majority of dependent members in WHHs are children. As raising a child is less costly than a dependent adult in the Vietnamese context, dependent members with many children might have minimal effect on the risk of poverty in WHHs.

The results in Table 2 also show a strong influence of education on poverty risk, and again this factor appears to be especially consequential in widow households. In the sample as a whole, a one-year increment in the grade of education completed by the household head reduces the odds of household poverty by 1.6 percentage points at mean values; in the sub-sample of widow households, the drop is 2.1 percentage points. Finally, age is a further protective factor against poverty, with each additional year of age-associated, at mean values, with a slight (but statistically significant) reduction in the likelihood of poverty (0.9 points in widow households and 0.6 points in all households). These results highlight the relatively high risks of poverty for older widows and those with lower levels of education.

The results on the locational variables show, first, that urban location is generally associated with a lower risk of poverty. However, this relationship is not statistically significant in the sub-sample of widow households. There are also substantial regional differences evident in the results, with households in the Mountainous and Northern Areas, the Coastal Central Region and the Central Highlands having a relatively high risk of poverty and relatively low-income levels. Outcomes for widows follow a similar pattern, although the poverty risk for widows in the Central Highlands is not significantly different from that in the Red River Delta. The results for widows in the Mountainous and Northern Areas (“North of the Middle” area) are of particular concern. Their widow households face a risk of poverty that is 10.0 percentage points higher than that experienced by their counterparts in the Red River Delta. Other households in this region are also, on average, economically deprived, but the relative disadvantage of widows appears to be particularly large.

Empirical results from the probit regression (in Table 2) are now compared with those from the logit regression (in the Appendix). We note that the empirical results under both estimation techniques largely remain the same. The vulnerability of widow households is consistently confirmed using these two regressions. Widowed households are more likely to fall into poverty, especially WHHs than others. This result aligns with findings from [5,21]. Other demographic characteristics also reveal the similarity between empirical results from the probit and logit regressions. Household size, years of education, and value of owned land reduce the probability of falling into poverty, especially in widow households. A geographical location such as mountainous or isolated areas and regions of ethnic minority (including Western North and North of the middle regions) is associated with a higher probability of poverty. These results are identical to [5,20,21].

Table 3 presents complementary results–showing the estimated coefficients on variables included in an OLS regression model of household income and household land value, respectively. The data in Columns 1 and 5 show, among other things, that widow households have lower household income (by 10.0% at mean values) than households where a widow is not present. However, their land holdings are not significantly different from non-widow households. The results in Columns 2 and 6 reveal that WHHs have similar incomes and land holdings to non-widow households, whilst other widow households tend to have lower incomes and hold more land. Thus, as also apparent in the results in Columns 3 and 7, WHHs tend to have more income but less land than other widow households. The results in Columns 4 and 8 suggest that WHHs have similar income and land asset levels as other FHHs.

**Table 3:** Determinants of household income and owned land value.

Variables	Household income is the dependent variable				Land value is the dependent variable			
	Model 1: All sample (1)	Model 2: All sample (2)	Model 3: Sub-sample Widow Households (3)	Model 4: Female-headed households (4)	Model 1: All sample (5)	Model 2: All sample (6)	Model 3: Sub-sample Widow Households (7)	Model 4: Female-headed households (8)
Widowed household	-0.10***				0.08			
	(0.02)				(0.14)			
Widow headed household		-0.01	0.28***	0.00		-0.15	-0.68*	-0.23
		(0.03)	(0.06)	(0.04)		(0.18)	(0.36)	(0.27)
Widow household where the head is not a widow		-0.24***				0.44**		
		(0.03)				(0.22)		
Household size (Equivalent scale)	0.37***	0.39***	0.52***	0.54***	0.03	0.00	-0.08	0.26**
	(0.01)	(0.01)	(0.02)	(0.02)	(0.06)	(0.06)	(0.14)	(0.13)
Number of absent members	-0.02	-0.02	-0.07	-0.03	0.01	0.02	-0.35	-0.24
	(0.02)	(0.02)	(0.05)	(0.04)	(0.13)	(0.13)	(0.31)	(0.29)
Dependency ratio (Equivalent scale)	-0.23***	-0.27***	-0.37***	-0.36***	0.50*	0.58*	-0.20	0.53
	(0.05)	(0.05)	(0.08)	(0.08)	(0.30)	(0.30)	(0.54)	(0.47)
Head’s educational attainment	0.09***	0.09***	0.07***	0.08***	0.05***	0.04***	-0.00	0.06*
	(0.00)	(0.00)	(0.01)	(0.00)	(0.02)	(0.02)	(0.04)	(0.03)
Head’s age	0.03***	0.03***	0.03***	-0.00	0.19***	0.19***	0.03	0.29***
	(0.00)	(0.00)	(0.01)	(0.01)	(0.03)	(0.03)	(0.06)	(0.05)
Head’s age squared	-0.00***	-0.00***	-0.00***	-0.00	-0.00***	-0.00***	-0.00	-0.00***
	(0.00)	(0.00)	(0.00)	(0.00)	(0.00)	(0.00)	(0.00)	(0.00)
Total injury times	-0.02***	-0.02***	-0.03**	-0.04***	0.03	0.03	0.04	0.08
	(0.01)	(0.01)	(0.01)	(0.02)	(0.05)	(0.05)	(0.09)	(0.07)
Share of wage in household income	-0.37***	-0.38***	-0.30***	-0.28***	0.91***	0.92***	0.95***	0.77***
	(0.02)	(0.02)	(0.05)	(0.04)	(0.07)	(0.07)	(0.15)	(0.14)
Large City^a^	0.33***	0.33***	0.35***	0.31***	0.37*	0.38**	1.21***	0.84**
	(0.02)	(0.02)	(0.06)	(0.05)	(0.19)	(0.19)	(0.41)	(0.35)
Medium City	0.25***	0.25***	0.20***	0.19***	0.28*	0.28*	0.86**	0.43
	(0.02)	(0.02)	(0.05)	(0.04)	(0.15)	(0.15)	(0.34)	(0.28)
Western North^b^	-0.51***	-0.51***	-0.36***	-0.11	-2.19***	-2.18***	-3.47***	-0.76
	(0.04)	(0.04)	(0.11)	(0.08)	(0.24)	(0.24)	(0.65)	(0.51)
Eastern North	-0.32***	-0.31***	-0.19**	-0.11*	-1.30***	-1.30***	-2.38***	-1.02**
	(0.03)	(0.03)	(0.07)	(0.06)	(0.19)	(0.19)	(0.45)	(0.42)
North of the Middle	-0.32***	-0.32***	-0.29***	-0.21***	-0.94***	-0.94***	-0.97**	-0.32
	(0.03)	(0.03)	(0.07)	(0.06)	(0.18)	(0.18)	(0.41)	(0.39)
South of the Middle	0.08***	0.08***	0.08	0.10*	-1.34***	-1.34***	-1.16***	-0.67*
	(0.03)	(0.03)	(0.07)	(0.06)	(0.19)	(0.19)	(0.40)	(0.38)
Highland	-0.06*	-0.06*	-0.12	-0.10	-0.72***	-0.71***	-1.55***	-0.69
	(0.04)	(0.04)	(0.09)	(0.07)	(0.22)	(0.22)	(0.58)	(0.50)
Eastern South	0.29***	0.29***	0.32***	0.31***	-2.13***	-2.13***	-1.71***	-1.85***
	(0.03)	(0.03)	(0.06)	(0.05)	(0.21)	(0.21)	(0.49)	(0.40)
Mekong Delta	0.22***	0.22***	0.28***	0.25***	-1.13***	-1.13***	-1.66***	-1.36***
	(0.03)	(0.03)	(0.06)	(0.05)	(0.16)	(0.16)	(0.39)	(0.35)
Constant	9.27***	9.29***	8.36***	9.58***	-5.89***	-5.99***	-0.40	-8.55***
	(0.10)	(0.10)	(0.27)	(0.19)	(0.95)	(0.95)	(2.09)	(1.92)
Observations	9,399	9,399	1,676	2,399	9,399	9,399	1,676	2,399
R-squared	0.402	0.404	0.521	0.530	0.078	0.078	0.093	0.088

Note: a) living in the rural area is the reference category; b) Red River Delta is the reference category; Robust standard errors in parentheses; ***, **, * is 1%, 5%, and 10% significant level, respectively.

The pattern in the results across Tables 2 and 3 indicates that at mean values and in the presence of controls for a range of demographic and other factors, being a WHH increases the risk of poverty. However, it has only a marginal effect on income. One possible explanation is that a relatively high proportion of WHHs are ’pushed over’ the poverty threshold by their lower income. At mean values, other widow households are associated with lower incomes than non-widow households but (as the results in Table 2 show) somewhat similar risks of poverty. This could show that a smaller proportion of these households’ incomes are close to the poverty threshold. The results on the land variables also suggest that WHHs are characterized by relatively low levels of land ownership, whilst the opposite is true of other widow households. Thus, the pattern of coefficients across the variables that measure household type in the income and poverty models could also reflect the influence that lands assets have on the chances of households being categorized as poor, as the results in Table 2 suggest the case.

The results in Table 3 for the various control variables follow a more consistent pattern. As was the case in the results of the poverty model, household size is positively related to income–and this relationship is relatively strong in widow households. Education has a measured positive impact on income, although the size of this effect is relatively weak in widow households. Age also has a positive effect on income in widows and other households. However, it is not positively related to land holdings in widow households (although it has substantial positive impacts in other households). Urban location is a further positive factor for household income, but, again, the strength of this influence is relatively small in widow households.

The dependency ratio is negatively related to income in widow and non-widow households. This result is somewhat at odds with the pattern of results in Table 3 (where the dependency ratio was not a statistically significant source of variation in poverty risk in widow households). Nevertheless, it adds to the questions about the underlying relationships. In the broad sample, the data in Tables 2 and 3 show that higher dependency is associated with lower income and higher poverty risk. In the sub-sample of widow households, this factor–which we can link to differences in women’s economic participation–tends to lower household income but not to the extent that it adds to the risk of being classified as poor. The results of the locational variables confirm that the Middle of the North region is relatively economically disadvantaged. Widow households in this region experience relatively low incomes, as do non-widow households. Land values are also lower, but not to the extent that they are in other northern areas. For example, the average land holding of widow households in the Middle of the North is 97 per cent lower than in the Red River region, but in the Western North, they are 347% lower.

The final results of this study–presented in Tables 4 and 5 –are on the risk of various types of resource insufficiency. Generally, these do not reveal significant differences between the widow and other households. However, where widows are exposed to poverty, the risks are highest for WHHs in the areas of water and housing insufficiency. WHHs have a chance of water insufficiency that is one percentage point higher than other widows at mean values; and a chance of housing insufficiency that is 1.5 percentage points higher. The results in these tables also confirm the vulnerability to poverty of households where the head has a low level of education, and they further illustrate the regional disparities in poverty risks. Widow households in the Eastern North appear especially vulnerable to food and foodstuff insufficiency.

**Table 4:** Determinants of household resource insufficiency, all households, probit marginal effects.

Variables	Form of insufficiency					
	Food	Foodstuff	Electricity	Water	Housing	Clothing
Widow headed household	-0.004	0.005	-0.003	0.001	0.002	-0.004
	(0.005)	(0.008)	(0.006)	(0.005)	(0.007)	(0.007)
Widow household where the head is not a widow	0.007	0.012	-0.009	-0.010	-0.016*	0.007
	(0.006)	(0.009)	(0.007)	(0.007)	(0.009)	(0.007)
Household size (Equivalent scale)	-0.006***	-0.004*	0.003	0.002	0.009***	-0.000
	(0.002)	(0.002)	(0.002)	(0.001)	(0.002)	(0.002)
Number of absent members	-0.006	-0.000	0.002	-0.002	0.001	-0.008
	(0.004)	(0.005)	(0.004)	(0.004)	(0.005)	(0.005)
Dependency ratio (Equivalent scale)	0.018**	0.024**	0.002	-0.015**	0.019*	0.039***
	(0.007)	(0.012)	(0.009)	(0.007)	(0.011)	(0.010)
Head’s educational attainment	-0.004***	-0.009***	-0.004***	-0.003***	-0.004***	-0.006***
	(0.000)	(0.001)	(0.001)	(0.000)	(0.001)	(0.001)
Head’s age	-0.001**	-0.004***	-0.002**	-0.002***	-0.003***	-0.002***
	(0.001)	(0.001)	(0.001)	(0.001)	(0.001)	(0.001)
Head’s age squared	0.000	0.000***	0.000*	0.000***	0.000**	0.000**
	(0.000)	(0.000)	(0.000)	(0.000)	(0.000)	(0.000)
Total injury times	0.000	0.004***	-0.001	0.001	0.002**	0.002*
	(0.001)	(0.001)	(0.001)	(0.001)	(0.001)	(0.001)
Large City^a^	-0.005	-0.035***	-0.024***	-0.001	0.005	-0.008
	(0.007)	(0.012)	(0.008)	(0.006)	(0.007)	(0.009)
Medium City	-0.004	-0.015**	-0.016***	-0.010**	-0.002	-0.008
	(0.004)	(0.007)	(0.006)	(0.005)	(0.006)	(0.006)
Western North^b^	0.037***	0.079***	0.038***	0.029***	0.003	0.043***
	(0.007)	(0.009)	(0.007)	(0.007)	(0.009)	(0.009)
Eastern North	0.032***	0.052***	0.037***	0.022***	0.015**	0.043***
	(0.007)	(0.009)	(0.006)	(0.006)	(0.008)	(0.008)
North of the Middle	0.019**	0.048***	0.014**	0.023***	0.010	0.039***
	(0.008)	(0.010)	(0.007)	(0.007)	(0.008)	(0.009)
South of the Middle	0.019**	0.011	-0.011	0.013*	0.007	0.017*
	(0.007)	(0.010)	(0.008)	(0.007)	(0.008)	(0.009)
Highland	0.026***	0.048***	-0.002	0.022***	0.023***	0.044***
	(0.008)	(0.010)	(0.009)	(0.007)	(0.008)	(0.009)
Eastern South	0.013*	0.000	0.005	0.005	0.017**	0.009
	(0.008)	(0.011)	(0.007)	(0.008)	(0.008)	(0.009)
Mekong Delta	0.003	-0.003	-0.010	0.018***	0.005	0.008
	(0.007)	(0.009)	(0.007)	(0.006)	(0.007)	(0.009)
Observations	9,330	9,317	9,276	9,206	9,186	9,267

Note: a) living in the rural area is the reference category; b) Red River Delta is the reference category; Robust standard errors in parentheses; ***, **, * is 1%, 5%, and 10% significant level, respectively.

**Table 5:** Determinants of household resource insufficiency, widowed households, probit marginal effects.

Variables	Form of insufficiency					
	Food	Foodstuff	Electricity	Water	Housing	Clothing
Widow headed household	0.000	0.005	0.005	0.010***	0.015***	-0.002
	(0.012)	(0.018)	(0.009)	(0.003)	(0.006)	(0.015)
Household size (Equivalent scale)	-0.007	-0.014**	0.001	-0.000	0.007***	0.001
	(0.005)	(0.007)	(0.003)	(0.002)	(0.003)	(0.004)
Number of absent members	0.007	-0.028	-0.031	-0.009	0.008	-0.013
	(0.009)	(0.020)	(0.022)	(0.009)	(0.006)	(0.016)
Dependency ratio (Equivalent scale)	-0.003	-0.015	-0.011	-0.019*	0.002	0.000
	(0.015)	(0.024)	(0.017)	(0.010)	(0.014)	(0.017)
Head’s educational attainment	-0.005**	-0.011***	-0.004**	-0.002**	-0.002*	-0.008***
	(0.002)	(0.003)	(0.002)	(0.001)	(0.001)	(0.003)
Head’s age	-0.003*	-0.005**	-0.000	-0.001	0.000	-0.004**
	(0.002)	(0.002)	(0.001)	(0.001)	(0.001)	(0.002)
Head’s age squared	0.000	0.000	-0.000	0.000	-0.000	0.000
	(0.000)	(0.000)	(0.000)	(0.000)	(0.000)	(0.000)
Total injury times	-0.012	0.003	-0.003	0.001	0.002	-0.000
	(0.008)	(0.005)	(0.004)	(0.001)	(0.001)	(0.003)
Large City^a^	0.002	-0.046	-	-0.002	-0.012	-0.016
	(0.016)	(0.029)		(0.009)	(0.012)	(0.024)
Medium City	0.003	-0.023	-0.000	-0.002	-0.003	-0.006
	(0.010)	(0.017)	(0.010)	(0.005)	(0.008)	(0.013)
Western North^b^	0.027	0.051*	0.007	0.014	-0.005	0.021
	(0.019)	(0.027)	(0.016)	(0.012)	(0.018)	(0.023)
Eastern North	0.037**	0.059***	0.021	0.016	0.004	0.035*
	(0.018)	(0.023)	(0.013)	(0.011)	(0.012)	(0.019)
North of the Middle	0.020	0.052**	-0.007	0.004	-0.006	0.027
	(0.017)	(0.023)	(0.015)	(0.011)	(0.014)	(0.019)
South of the Middle	0.019	0.013	-0.022	0.010	0.008	0.030
	(0.017)	(0.023)	(0.015)	(0.010)	(0.011)	(0.019)
Highland	0.024	0.051*	-0.008	0.016	0.025*	0.037*
	(0.017)	(0.026)	(0.017)	(0.012)	(0.013)	(0.020)
Eastern South	0.009	0.016	-0.016	0.002	0.020	-0.013
	(0.017)	(0.026)	(0.019)	(0.011)	(0.013)	(0.024)
Mekong Delta	-0.004	0.001	-0.025	0.016	0.006	0.002
	(0.016)	(0.022)	(0.016)	(0.010)	(0.010)	(0.019)
Observations	1,663	1,661	1,450	1,639	1,637	1,649

Note: a) living in the rural area is the reference category; b) Red River Delta is the reference category; Robust standard errors in parentheses; ***, **, * is 1%, 5%, and 10% significant level, respectively.

## 5. Concluding remarks

This study has contributed an analysis of the economic circumstances of Vietnamese widows, adding to a very small extant literature on the incidence of poverty amongst widows and the factors affecting this risk. The results from our analysis of the VHLSS 2018 data confirm that widows are relatively vulnerable to poverty. This risk is relatively large for widows that are household heads and, in this way WHHs are similar to other FHHs in Vietnam. Higher levels of education, older age, urban location, and a larger household size also appear to provide some Vietnamese widows with protection against the risk of poverty. However, the disparity level in widows’ poverty risks is high, with an especially strong regional variation. The high poverty rate amongst widows in the Middle North regions is particularly concerning. These findings indicate that, despite strong efforts at developing state institutions for gender equality, additional efforts are needed to address the disadvantaged economic position of key groups of women.

This study has also contributed some fresh insights into the economic situation of female-headed households in Vietnam. Whilst some prior studies have shown that FHHs in Vietnam are better off than male-headed households [2], we find that widow-headed households are relatively disadvantaged, experiencing a relatively high risk of poverty. Thus, it would seem that when the route into female headship is through widowhood, at least, the risk of poverty for Vietnamese women is real.

The study has focused only on statistical patterns in existing data sets. It will be important that future work involves the collection of new quantitative data and that complementary qualitative investigations take place. On the quantitative side, new measures are needed to assess the distribution of resources within the households in that widows reside. Our results suggest that households that include a widow who is not the head are not as economically disadvantaged as WHHs; however, it is not clear whether the widows in these households are protected from poverty. Our results also show, for example, that widows who live in the Northern Mountainous areas experience high poverty risks. However, we cannot be sure about the institutional or other factors that contribute to these risks. There is a need, therefore, to keep adding to the informational base on widows’ economic circumstances, perhaps using the current study results as a guide to the key areas of policy concern.

## Supporting information

S1 Table Determinants of household poverty, all Vietnamese households, widow households and female-headed households, logit marginal effects. (DOCX)
