# Supplementary material for: The economic circumstances of widows in Vietnam
Source: PLoS One. 2023 May 10;18(5):e0285595. doi: 10.1371/journal.pone.0285595 (PMC10171680; doi:10.1371/journal.pone.0285595)
Supplement: S1 Table — (DOCX) [file pone.0285595.s001.docx]

**S1 Table: Determinants of household poverty, all Vietnamese households, widow households and female-headed households, logit marginal effects**

|  | All sample | All sample | Widow Households^1^ | Female-headed households^2^ |
| --- | --- | --- | --- | --- |
| Widowed household | 0.024*** |  |  |  |
|  | (0.007) |  |  |  |
| Widow headed household |  | 0.028*** | 0.036 | 0.022 |
|  |  | (0.009) | (0.022) | (0.016) |
| Widow household where the head is not a widow |  | 0.017 |  |  |
|  |  | (0.013) |  |  |
| Household size (Equivalent scale) | -0.013*** | -0.012*** | -0.030*** | -0.025*** |
|  | (0.003) | (0.003) | (0.010) | (0.007) |
| Number of absent members | -0.002 | -0.003 | 0.037 | 0.036* |
|  | (0.009) | (0.009) | (0.025) | (0.019) |
| Dependency ratio (Equivalent scale) | 0.047*** | 0.046*** | -0.004 | 0.022 |
|  | (0.014) | (0.014) | (0.028) | (0.023) |
| Head’s educational attainment | -0.016*** | -0.016*** | -0.021*** | -0.020*** |
|  | (0.001) | (0.001) | (0.004) | (0.002) |
| Head’s age | -0.006*** | -0.006*** | -0.009*** | -0.002 |
|  | (0.001) | (0.001) | (0.003) | (0.003) |
| Head’s age squared | 0.000*** | 0.000*** | 0.000** | 0.000 |
|  | (0.000) | (0.000) | (0.000) | (0.000) |
| Total injury times | 0.007*** | 0.007*** | 0.008 | 0.009** |
|  | (0.002) | (0.002) | (0.005) | (0.004) |
| Land value (Log) | -0.003*** | -0.004*** | -0.005*** | -0.004*** |
|  | (0.000) | (0.000) | (0.001) | (0.001) |
| Large City^a^ | -0.082*** | -0.083*** | -0.048 | -0.076*** |
|  | (0.017) | (0.017) | (0.034) | (0.027) |
| Medium City | -0.050*** | -0.051*** | -0.029 | -0.053*** |
|  | (0.010) | (0.010) | (0.022) | (0.018) |
| Western North^b^ | 0.085*** | 0.086*** | 0.072** | 0.053** |
|  | (0.012) | (0.012) | (0.033) | (0.026) |
| Eastern North | 0.070*** | 0.070*** | 0.070** | 0.042* |
|  | (0.011) | (0.011) | (0.027) | (0.023) |
| North of the Middle | 0.064*** | 0.064*** | 0.098*** | 0.074*** |
|  | (0.012) | (0.012) | (0.028) | (0.023) |
| South of the Middle | 0.014 | 0.014 | 0.004 | -0.019 |
|  | (0.012) | (0.012) | (0.027) | (0.022) |
| Highland | 0.038*** | 0.038*** | 0.004 | 0.013 |
|  | (0.013) | (0.013) | (0.035) | (0.027) |
| Eastern South | -0.043*** | -0.043*** | -0.061* | -0.077*** |
|  | (0.016) | (0.016) | (0.036) | (0.028) |
| Mekong Delta | -0.014 | -0.014 | -0.024 | -0.061*** |
|  | (0.012) | (0.012) | (0.027) | (0.023) |
|  |  |  |  |  |
| Observations | 9,399 | 9,399 | 1,676 | 2,399 |
| Note: a) living in the rural area is the reference category; b) Red River Delta is the reference category; Robust standard errors in parentheses; ***, **, * is 1%, 5%, and 10% significant level, respectively.  ^1^ is a sub-sample of widow households where at least one widow lives.  ^2^ is a sub-sample of female-headed households. | | | | |
